# GWAS OF BRAIN ENDOPHENOTYPES: ASSOCIATION OF AUTOPSY-BASED LEWY BODY COUNTS WITH MOVEMENT DISORDER RISK LOCI

**DOI:** 10.1093/geroni/igad104.2234

**Published:** 2023-12-21

**Authors:** Steven Edland, Fernanado Hernandez, Chanond Nasamran, Kathleen Fisch, Lon White

**Affiliations:** University of California San Diego, San Diego, California, United States; University of California San Diego, San Diego, California, United States; University of California San Diego, San Diego, California, United States; University of California, San Diego, San Diego, California, United States; Pacific Health Research and Education Institute, Honolulu, Hawaii, United States

## Abstract

We performed a genome wide association study (GWAS) of neocortical Lewy body density. Case material were 308 autopsies of Japanese-American men (mean age at death 87.5 years; 11% with mild, and 30% with severe cognitive impairment prior to death). Lewy bodies were assessed by manual count of alpha-synuclein stained neocortical sections from the frontal, temporal, parietal, and occipital lobes. Genetic association was tested by linear models controlling for age at death assuming an additive genotypic effect. Top hits include a locus on chromosome 1q32 previously associated with amyotrophic lateral sclerosis (p=5.8e-8), intronic variants on the dopaminergic neuron axonal guidance gene EPHB1 previously associated with Parkinson’s disease (PD) (p=8.4e-6), and a locus containing two independent intron 1 variants on CSMD1, a known risk factor for autosomal dominant PD (p=1.5e-6). Complement inhibitor protein CSMD1 is a type 1 transmembrane cell adhesion molecule implicated in axonal guidance and the development, maintenance, and pruning of synapses in the adult brain. Intermediate hits include genes encoding cell adhesion molecules and a second complement inhibitor implicated in the regulation of synapses (TRAPPC9). This converging evidence points to cell adhesion proteins and complement mediated synaptic regulation in the etiology of Lewy body lesions.

